# Hormonal and inflammatory modulatory effects of hesperidin in hyperthyroidism-modeled rats

**DOI:** 10.3389/fimmu.2023.1087397

**Published:** 2023-03-20

**Authors:** Mahmoud Ashry, Hussam Askar, Manar M. Obiedallah, Ahmed Hussuin Elankily, Doaa Galal El-Sahra, Gamal Zayed, Mohamed A. Mustafa, Sawsan Abd El-Maksoud El-Shamy, Somaia A. Negm, Marwa A. El-Beltagy, Khaled G. Abdel-Wahhab, Antoaneta Ene

**Affiliations:** ^1^ Zoology Department, Faculty of Science, Al-Azhar University, Assiut, Egypt; ^2^ Institute of Chemical Technology, Ural Federal University, Ekaterinburg, Russia; ^3^ Department of Pharmaceutics, Faculty of Pharmacy, Assiut University, Assiut, Egypt; ^4^ Medical Surgical Nursing Department, Faculty of Nursing, Modern University for Technology and Information, Cairo, Egypt; ^5^ Department of Pharmaceutics and Pharmaceutical Technology, Faculty of Pharmacy, Al-Azhar University, Assiut, Egypt; ^6^ Faculty of Applied Health Sciences, New Assiut Technological University, Assiut, Egypt; ^7^ Basic Centre of Science, Misr University for Science and Technology, Giza, Egypt; ^8^ Biology Department, Basic Centre of Science, Misr University for Science and Technology, Giza, Egypt; ^9^ Faculty of Applied Health Science Technology, Misr University for Science and Technology, Giza, Egypt; ^10^ Biochemistry Department, Faculty of Veterinary Medicine, Suez Canal University, Ismailia, Egypt; ^11^ Medical Physiology Department, National Research Centre, Giza, Egypt; ^12^ INPOLDE Research Center, Department of Chemistry, Physics and Environment, Faculty of Sciences and Environment, Dunarea de Jos University of Galati, Galati, Romania

**Keywords:** hyperthyroidism, L-Thyroxine, anti-inflammatory, hesperidin, rat

## Abstract

The goal of the current study was to investigate the hormonal modulatory efficiency of hesperidin, through its regulatory potential of immunological, inflammatory, and/or antioxidant changes in on hyperthyroidism modeled adult female albino rats. Both normal and hyperthyroidism modeled rats (140-160g) were randomly divided into four groups (10 animals each) as follows: 1) healthy animals were daily ingested with saline for six weeks, and served as control group, 2) healthy animals were intraperitoneally injected with hesperidin (50 mg/kg/day) for a similar period, 3) hyperthyroidism-modeled animals without any treatment acted as positive control, and 4) hyperthyroidism-modeled animals were treated intraperitoneally with hesperidin for a similar period. The findings showed that hesperidin significantly modulated hyperthyroidism deteriorations, this was evidenced by a remarkable decline in serum T4, FT4, T3, FT3, TNF-α, IL1β-, IL4-, IL-6, and IL-10 levels, with a minor increase in TSH and significant raise in CD4+ level. Similarly, valuable improvement was observed in the oxidative status; serum SOD, GPx, CAT, and GSH levels were dramatically enhanced, associated with remarkable drop in MDA and NO levels. Also, hesperidin demonstrated nephro-hepatoprotective and anti-atherogenic potential, this was achieved from the notable reduction in ALAT and ASAT activities as well as urea, creatinine, cholesterol, and triglyceride close to the corresponding values of healthy group. These findings were supported by histological and immunohistochemical ones that showed a notable decrease in the expression of the calcitonin antibody. In conclusion, hesperidin possesses anti-hyperthyroidism, immunoinflammatory regulatory, and antioxidant activities that evidenced from the improvement of physio-architecture of the thyroid gland, reduction of inflammation and restoration of the impaired oxidative stress. This effect might be mechanized through immunological, inflammatory, apoptotic, and/or antioxidant modulatory pathways.

## Introduction

One of the most crucial components of the human body is the thyroid gland, which controls the majority of its physiological processes (1). Thyroid hormones (Triiodothyronine and thyroxine) are necessary for growth, development, and the control of energy metabolism *via* their impact on protein, carbohydrate, and lipid metabolism. In addition, these hormones regulate numerous crucial regulatory hormones, including catecholamines and insulin (2–4). Any thyroid disorder can alter the production of thyroid hormones, and result in several pathophysiological disorders throughout the body (1). According to Costilla et al. (5), the endocrine condition, hyperthyroidism, is defined as an excessive release of thyroid hormones (T3 and T4), which raise basal metabolic rate and oxygen consumption in a variety of tissues (2, 3). The most common cause of hyperthyroidism is Graves’ disease, followed by toxic nodular goiter; however, autonomous functioning thyroid nodules or toxic nodular goiter are less frequent ones (6). A hypermetabolic condition brought on by hyperthyroidism is linked to varying levels of oxidative stress in the body; in hyperthyroid rats, oxidative stress-related damages to proteins, lipids, and DNA have been reported (3).

Adverse reactions can occur with anti-hyperthyroid drugs, mostly severe liver damage; however, many are life threatening, and their management is complex, and currently a key debate amongst endocrinologists (7). A lot of research has been done recently on plants that are rich in natural phytochemicals such polyphenols, tocopherols, flavonoids, alkaloids, tannins, carotenoids, and terpenoids because of their significant immunomodulatory, anti-carcinogenic, and antioxidant capabilities (8). Hesperidin (3’, 5, 7-trihydroxy-4’-methoxy flavanone-7-6-O-Lrhamnosyl-D-glucose) is a naturally occurring flavonoid that is widely distributed in plants and fruits. (9). It is an inexpensive byproduct of the citrus industry and one among the most significant bioflavonoids in sweet orange and lemon (10). Hesperidin has a variety of therapeutic benefits, including anti-inflammatory, antioxidant, and allergy properties (11). In rats’ carcinogenesis models, hesperidin was found to have anti-carcinogenic properties in the urinary bladder, colon, tongue, and oesophagus (12). Another naturally occurring flavonoid, hesperidin aglycone (3’, 5, 7-trihydroxy-4’-methoxy flavanone), has been shown to have intriguing therapeutic potential in neuropathological diseases (9).

As hyperthyroidism has been linked to a higher incidence of pathophysiological complications, and hesperidin possesses antioxidant, antiadipogenic, anti-inflammatory, anti-hyperlipidemic, and anti-apoptotic agent; therefore, the goal of this study was to investigate the anti-hyperthyroidism, immune-regulatory, and/or anti-inflammatory effectiveness of hesperidin and the possible mechanisms on hyperthyroidism modeled rats.

## Materials and methods

### Chemicals

L-Thyroxine (levothyroxine) was purchased from Sigma Aldrich, USA. Hesperidin was obtained from Sedico Pharmaceutical Company, 6^th^ October City, Cairo, Egypt.

### Animals

Adult female Wistar albino rats (140-160g) were obtained from Animal Colony, National Research Centre, Giza, Egypt. One week before starting the experiment, the animals were housed in suitable plastic cages for acclimatization. Excess tap water and standard rodent pellets were always available. All animals received human care in compliance with the standard institutional criteria for the care and use of experimental animals according to the guidelines of the ethical committee of Faculty of Science, Al-Azhar University.

### Induction of hyperthyroidism

After acclimatization, hyperthyroidism was induced as previously described by Hwang et al. (13). In brief, a suitable number of the animals was subcutaneously injected with levothyroxine (0.3mg/kg/day). Blood samples were withdrawn every 2 weeks and tested for T4, FT4, T3, FT3 and TSH; animals without elevated thyroid profile were excluded.

### Experimental design

After induction of hyperthyroidism, both healthy and hyperthyroidism-modeled animals were randomly divided into four groups (10 animals each) as in the following **Table 1**.

**Table 1:** Experimental design.

Animals’ group	Treatment
Group 1	Healthy animals were daily ingested with saline for six weeks and served as control group.
Group 2	Healthy animals were intraperitoneally injected with 50 mg/kg/day (14) of hesperidin suspension (in saline) for a similar period.
Group 3	Untreated hyperthyroidism-modeled animals (13).
Group 4	Hyperthyroidism-modeled animals treated with hesperidin suspension (50 mg/kg/day, ip) for a similar period.

### Blood and tissue sampling

At the end of the experimental period, all animals were fasted overnight and weighed; then post-anesthesia, blood specimens (3ml/animal) were withdrawn, left to clot, and cool-centrifuged. The sera were separated, divided into aliquots, and stored at -80°C till hormonal and biochemical measurements could be carried out as soon as possible. After blood collection, the animals were sacrificed soon *via* sudden decapitation, and the thyroid of each animal was dissected out with a part of the trachea, soaked in formalin-saline (10% v/v) buffer for histopathological and immunohistochemical processing and microscopic examination.

### Thyroid hormonal profile

Thyroid serum hormonal profile (total T4, freeT4, total T3, free T3, and TSH levels) was determined using rats’ reagent ELISA-kits (SL-1057Ra, SL-1079Ra, SL-1027Ra, SL-1047Ra and SL-1037Ra, respectively) purchased from Sunlong Biotech Co, Hang Zhou, China.

### Oxidative stress and antioxidant assay

Serum values of GSH, GPx, NO, MDA, CAT and SOD were measured using rats’ reagent ELISA-kits (SL-8577Ra, SL-1033Ra, SL-0531Ra, SL-8612Ra, SL-5986Ra and SL-8178Ra, respectively) purchased from Sunlong Biotech Co, Hang Zhou, China.

### Immunoinflammatory and apoptotic markers

Serum level of TNF-α, IL-1β, IL-4, IL-6, IL-10 and CD4 was measured using rats’ reagent ELISA-kits (SL-0403Ra, SL-0402Ra, SL-1154Ra, SL-1158Ra, SL-1144Ra and SL-1215Ra, respectively) purchased from Sunlong Biotech Co, Hang Zhou, China.

### Biochemical determinations

Serum ASAT and ALAT activity, as well as urea, creatinine, total cholesterol, triglycerides, LDL-cholesterol, HDL-cholesterol, and glucose were estimated spectrophotometrically using reagent kits (UR-2110, CR1250, AL1031, AS1061, CH1220, TR2030, CH1230, CH1230 and GL1320, respectively) obtained from Biodiagnostic Co., Dokki, Giza, Egypt.

### Histopathology

The histological preparations were carried out as described by Bancroft and Stevens (15). Briefly, thyroid tissues were sliced to 3-4 mm thick, dehydrated in graded concentrations of ethanol, cleared in xylene, and dyed with Hematoxylin and Eosin stain and the sections were examined microscopically.

### Immunohistochemistry

Immunohistochemistry staining was carried out on paraffin sections and mounted on positively charged slides by using avidin-biotin-peroxidase complex (ABC) method (16). The sections were incubated with anti-rabbit Calcitonin Receptor Antibody (ThermoFisher Scientific, Cat# PA1-84457, Polyclonal, Dil.: 1:1000), then the reagents required for ABC method (Vectastain ABC-HRP kit, Vector laboratories) were added. Marker expression was labeled with peroxidase and colored with diaminobenzidine (DAB, Sigma-USA) to detect antigen-antibody complex. Negative controls were included using nonimmune serum in place of the primary or secondary antibodies. Finally, the stained sections were examined microscopically.

### Scoring of immunohistochemical findings or area percentage *(Specific area/antibody)*

The areas that displayed a positive brown-immunostaining were selected for evaluation (regardless the strength of staining) using Leica scoring program, where measurement units (pixels) are converted into actual micrometer units. Calcitonin immune-stained-sections were microscopically (Light Leica Microscopy) measured as area percentage in a standard representative six fields using 400 x magnifications.

### Statistical analysis

The obtained data were subjected to one-way ANOVA followed by Duncan multiple *post hoc* tests at p ≤ 0.05 (17) using a statistical analysis system (SAS) program software; copyright (c) 1998 by SAS Institute Inc., Cary, NC, USA.

## Results

The current study showed that the thyroid hormone profile didn’t change after pure hesperidin was administered to healthy animals. It is interesting to note that hesperidin treatment of the hyperthyroidism-model animals resulted in a significant improvement in thyroid hormone levels. This was accomplished by a marked downregulation of total T4, free T4, total T3, and free T3, matched with a slight upregulation of TSH level toward the corresponding levels of healthy animals’ group (**Table 2**). Additionally, rats with induced-hyperthyroidism displayed severe abnormalities in serum oxidative status, as shown by a marked decrease in the values of the antioxidants (GSH, SOD, CAT, and GPx) and a correspondingly significant rise in the levels of the oxidative markers (MDA and NO) when compared to the healthy control group (**Table 3**).

**Table 2:** Serum T3, FT3, T4, FT4 and TSH levels of healthy and hyperthyroidism -treated animals’ groups as compared to control group.

	Control	Hesperidin	Hyperthyroidism	Hyper ~ Hesperidin
T3 (ng/ml)	95.13 ± 5.3	93.86 ± 10.11	199.33 ± 13.05^*^	118.25 ± 8.05^#^
FT3 (pg/ml)	3.28 ± 0.47	3.24 ± 0.6	5.97 ± 0.38^*^	3.91 ± 0.31^#^
T4 (ug/dl)	6.40 ± 0.72	6.09 ± 0.31	16.56 ± 0.76^*^	9.8 ± 1.68^#^
FT4 (ng/dl)	2.1 ± 0.25	1.95 ± 0.9	4.7 ± .0.44^*^	2.99 ± 0.71^#^
TSH (uIU/mL)	1.09 ± 0.09	1.13 ± 0.08	0.16 ± 0.02^*^	0.71 ± 0.23^#^

Data are presented as mean ± standard error; data were subjected to one-way ANOVA followed by post-hoc test (Duncan) at p≤ 0.05. Symbol * is significantly different from control group, while symbol # is significantly different from hyperthyroidism group.

**Table 3:** Serum oxidant (MDA and NO) and antioxidant (GSH, SOD, GPx and CAT) markers of healthy and hyperthyroidism treated animals’ groups as compared to control group.

	Control	Hesperidin	Hyperthyroidism	Hyper ~ Hesperidin
MDA (pg/mL)	22.8±12.6	223.7±9.9	660.4±14.5^*^	341±3.9^#^
NO (µmol/L)	7.6±0.3	7.3±0.79	25.4±2.01^*^	12.12±2.2^#^
GSH (ng/mL)	11.73±0.68	13.11±0.35	5.15±0.41^*^	9.2±0.71^#^
SOD (U/L)	2.2±0.09	2.3±0.08	0.84±0.03^*^	1.5±0.07^#^
GPx (U/L)	960.5±58.3	986.3±28.6	426.3±56.3^*^	758±90.3^#^
CAT (U/L)	8.44±0.81	9.5±0.71	3.2±0.55^*^	7.2 ±0.67^#^

Data are presented as mean ± standard error; data were subjected to one-way ANOVA followed by post-hoc test (Duncan) at p≤ 0.05. Symbol * is significantly different from control group, while symbol # is significantly different from hyperthyroidism group.

**Figure 1:**
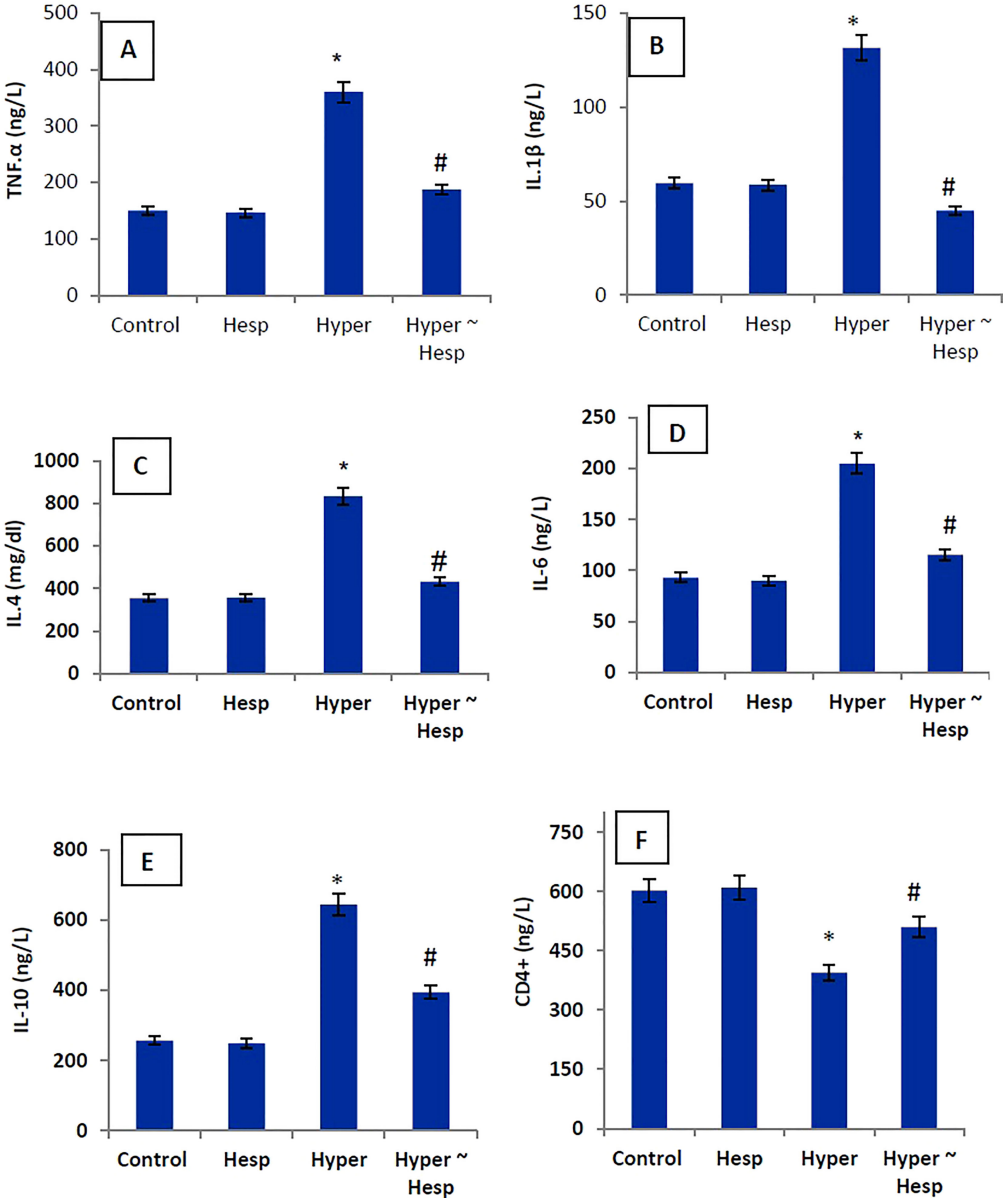
**(A–F)** Serum TNF-α, IL-1β, IL-4, IL-6, IL-10 and CD4+ levels of healthy and hyperthyroidism-treated animals’ groups as compared to control group. Data are presented as mean ± standard error; data were subjected to one-way ANOVA followed by *post-hoc* test (Duncan) at p≤ 0.05. Symbol * is significantly different from control group, while symbol # is significantly different from hyperthyroidism group.

The level of the pro-inflammatory markers (TNF-α, IL-1, IL-4, IL-6, and IL-10) in the hyperthyroidism group of rats was much higher than that of the healthy animals, whereas the level of the apoptotic marker (CD4+) was noticeably lower. Interestingly, treatment of hyperthyroidism-modeled group with hesperidin therapy dramatically decreased blood levels of TNF-α, IL-1, IL-4, IL-6, and IL-10 while sharply increasing CD4+ level close to healthy controls (**Figure 1**).

Hesperidin administration to healthy rats did not affect their liver or kidney functions, indicating that it had no harmful effects on either organ. In contrast, inducing hyperthyroidism caused a significant disturbance in liver and kidney function markers, as evidenced by the clearly elevated ALAT and ASAT activities, along with clearly elevated urea and creatinine levels when compared to healthy animals’ group. Treatment of hyperthyroidism-modeled animals with hesperidin considerably improved the impairment of the hepatic and renal systems, around the healthy group values (**Table 4**).

**Table 4:** Activities of serum ASAT and ALAT, and levels of serum urea, creatinine and glucose of healthy and hyperthyroidism-treated animals’ groups as compared to control group.

	Control	Hesperidin	Hyperthyroidism	Hyper ~ Hesperidin
ALAT (U/L)	51.5 ± 1.88	46.8 ± 2.91	72.13 ± 1.96^*^	52.3 ± 4.63^#^
ASAT (U/L)	72.13 ± 4.01	67.13 ± 2.67	91.5 ± 4.61^*^	74.25 ± 4.91^#^
Urea (mg/dl)	43.7 ± 2.5	42.3 ± 1.38	59.63 ± 1.96^*^	44.65 ± 2.09^#^
Creatinine (mg/dl)	0.53 ± 0.13	0.59 ± 0.11	1.41 ± 0.9^*^	0.70 ± 0.12^#^
Chol (mg/dl)	126 ± 4.3	117 ± 13.5	180.6 ± 29^*^	112.5 ± 21.6^#^
Trigl (mg/dl)	163.6 ± 41.1	156.6 ± 27	249 ± 39.2^*^	166.7 ± 25.5^#^
HDL-c (mg/dl)	45 ± 1	45.3 ± 2.08	34.3 ± 2.1^*^	42.7 ± 1.7^#^
LDL-c (mg/dl)	85 ± 7.9	85 ± 7.9	125 ± 10.5^*^	95.5 ± 5.8^#^
Glucose (mg/dl)	98.2 ± 4.7	98.7 ± 10.1	137.6 ± 8.2^*^	111.7 ± 9.8^#^

Data are presented as mean ± standard error; data were subjected to one-way ANOVA followed by post-hoc test (Duncan) at p≤ 0.05. Symbol * is significantly different from control group, while symbol # is significantly different from hyperthyroidism group.

The serum lipid profile and glucose levels of hesperidin-treated healthy rats were also unaffected. On the other hand, induced-hyperthyroidism resulted in the onset of atherosclerosis, as evidenced by the marked increase in serum levels of total cholesterol, triglycerides, LDL cholesterol, and glucose together with a pronounced decrease in HDL cholesterol. Post-treatment of hyperthyroidism-modeled rats with hesperidin significantly increased the levels of atherosclerotic markers as well as glucose level when compared to hyperthyroidism-modeled animal (**Table 4**).

### Histopathological findings

**Figure 2:**
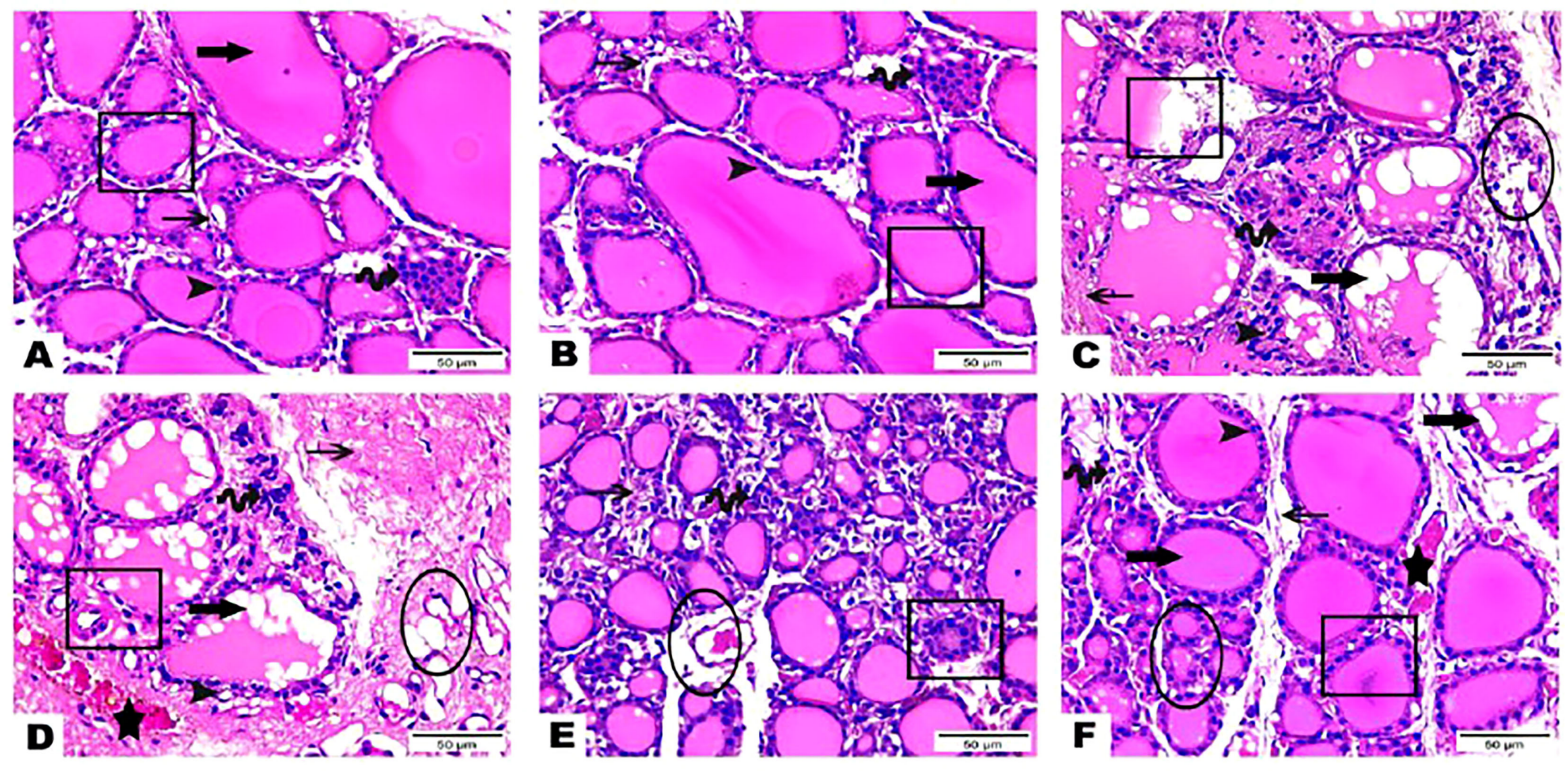
**(A–F)** Photomicrographs of thyroid gland sections of both **(A)** (G 1) and **(B)** group (G 2) displayed the standard histological architecture of its functional units; thyroid follicles (cube); these follicles are lined with simple cuboidal follicular epithelial cells that marked through eosinophilic cytoplasm and round, hyperchromatic nuclei (arrowhead). These cells encircle a central lumen packed with a consistent, pink colloid (thick arrow). Parafollicular C cells are represented as clusters of polygonal shape adjacent to thyroid follicles (wave arrow). Notice thin intra lobular fibrous connective tissue in between thyroid follicles (thin arrow). Thyroid gland sections **(C–E)** of (G 3) highlighted various degenerative changes along the thyroid gland; thyroid follicles detected with large, as well as small pleomorphic appearance and a marked deteriorated structure with desquamated epithelium in others (cubes). Obvious vacuolations with few colloid quantities are observed (thick arrows). Follicular and parafollicular cells were detected in high numbers and polygonal apoptotic shapes (arrowheads, wave arrows, respectively). Intense amount of fibrous connective tissue dispersed in between thyroid follicles (thin arrows). Blood vessels emphasized increase in number, degeneration, epithelial desquamation (circles), and congestion (stars). Thyroid gland section **(F)** of (G 4) exhibited obvious improvement in the thyroid gland structure; some thyroid follicles existed in normal assembly (cube) and others remarked small size (circle); colloid appeared mostly in a homogenous shape and few vacuolated ones (thick arrow). Intralobular connective tissue, follicular cells as well as para-follicular cells were presented in their normal structure (thin arrow, arrowhead, wave arrow; respectively). Notice congested blood vessels (star). (Hematoxylin & Eosin Stain, Magnification Power= x400 & Scale Bar= 50μm).

**Figure 3:**
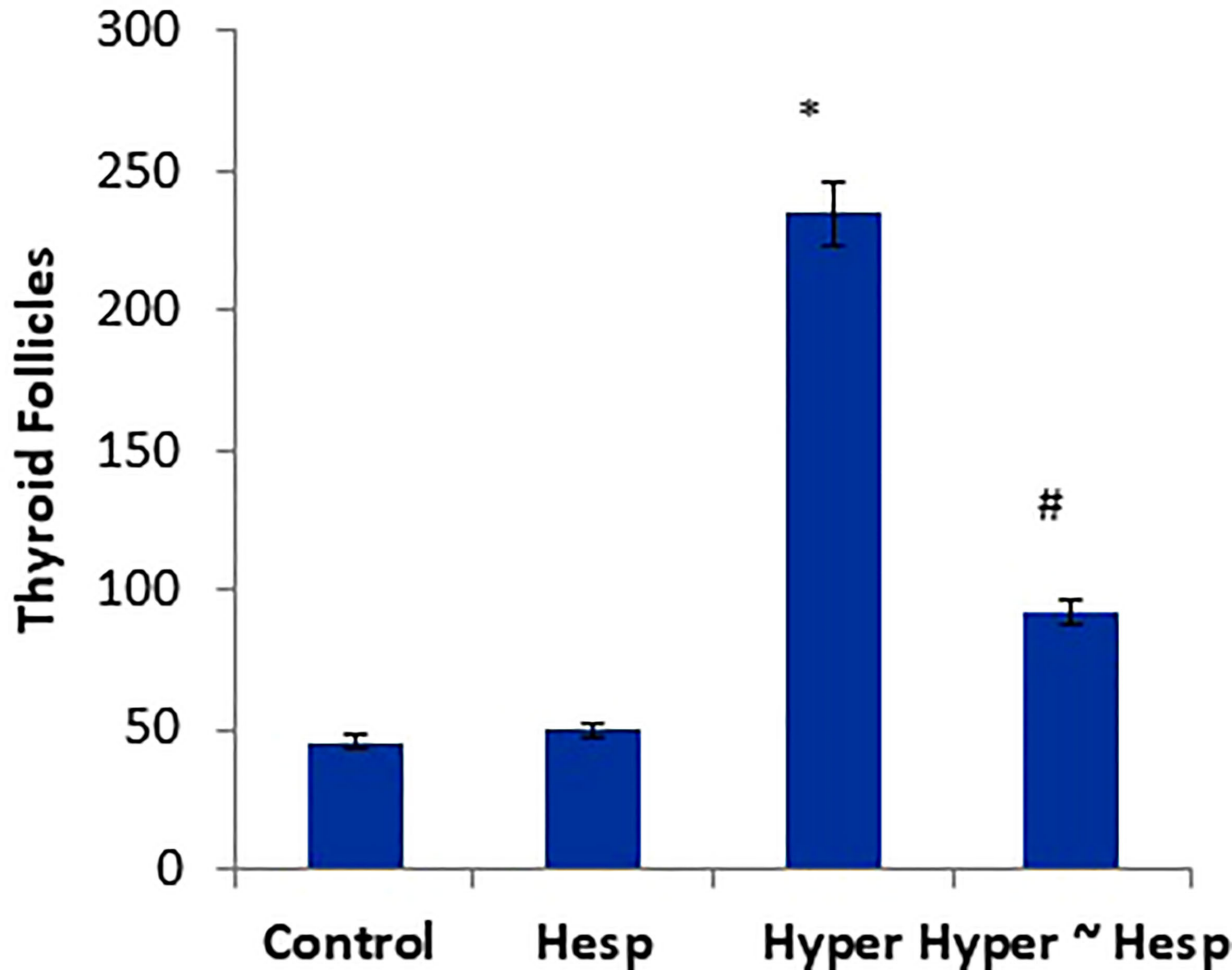
Scoring of thyroid follicles of healthy and hyperthyroidism treated animals’ groups as compared to control group. Data are presented as mean ± standard error and they were subjected to one-way ANOVA followed by *post-hoc* test (Duncan) at p≤ 0.05. Symbol * is significantly different from control group, while symbol # is significantly different from hyperthyroidism group.

The histological observations of the present study revealed that both control, and hesperidin-treated healthy animals’ groups showed normal thyroid architectures as they displayed typical histological architecture of the functional thyroid follicles that are lined with straightforward cuboidal follicular epithelial cells with eosinophilic cytoplasm and round, hyperchromatic nuclei. In contrary, hyperthyroidism-modeled animals highlighted various degenerative changes along the thyroid gland; this change was in form of large thyroid follicles, small pleomorphic appearance, and a marked deteriorated structure with desquamated epithelium in others; also, obvious vacuolations with few colloid quantities were observed; high number and polygonal apoptotic shapes of follicular and para-follicular cells were present; intense amount of fibrous connective tissue dispersed among thyroid follicles; blood vessels emphasized increase in number, degeneration, epithelial desquamation, and congestion. In a trusting manner, treatment of hyperthyroidism with hesperidin resulted in a remarkable improvement in the thyroid architecture; as some thyroid follicles were present in a normal assembly and others were noticeably tiny; the colloid largely had a homogenous shape and only a few vacuolated ones; also, follicular, and para-follicular cells of the intralobular connective tissue were shown in their typical number and configuration with clogged blood arteries in the figures (**Figures 2**, **3**).

### Immunohistochemistry findings

**Figure 4:**
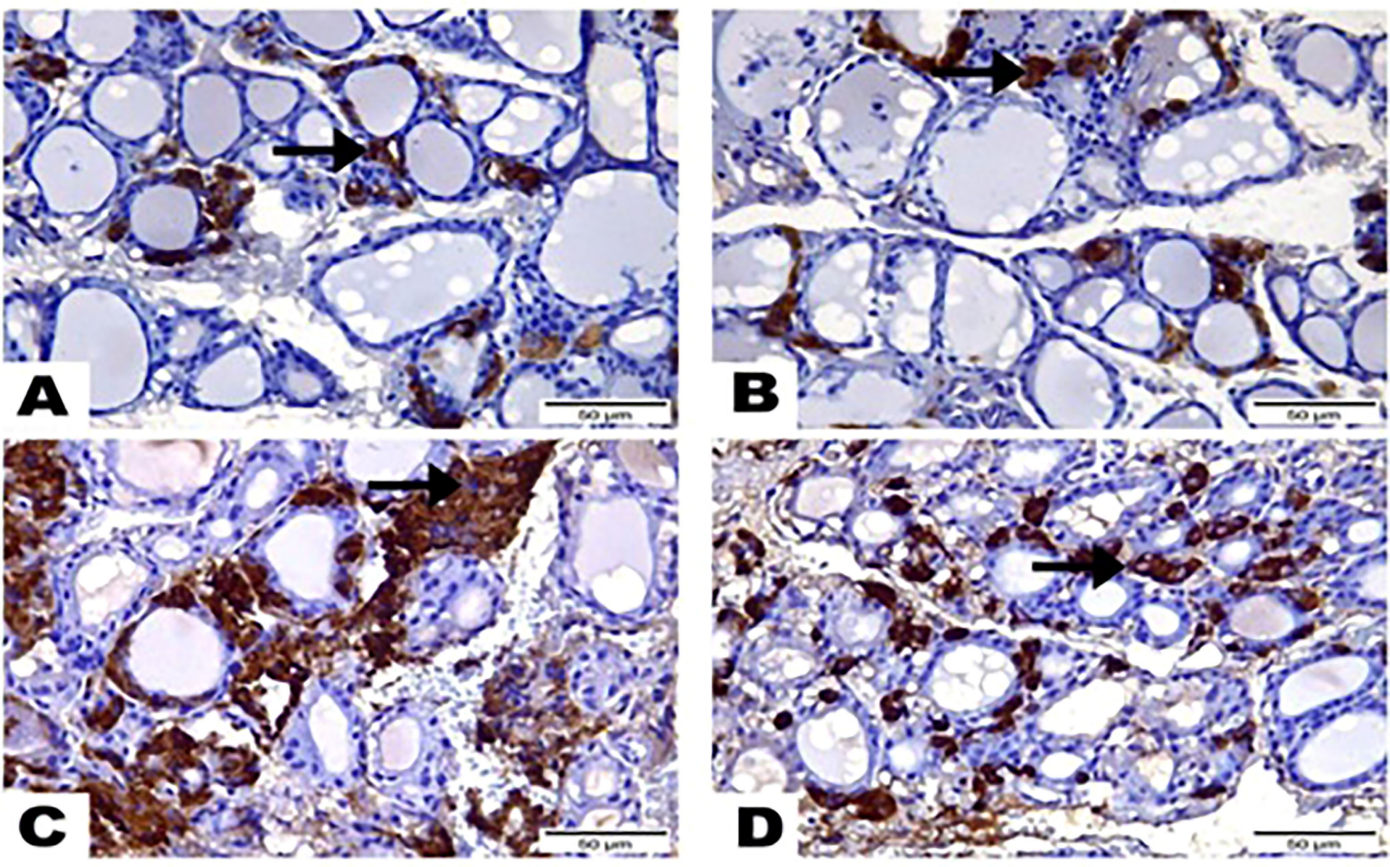
Photomicrographs displayed the expression of Calcitonin-Antibody, **(A)** in the thyroid gland section of (G1) expressed in a few amounts to Calcitonin Antibody (arrow), while greatest most para-follicular cells identified as blue negative nucleus. **(B)** Thyroid gland section of (G2) marked also little reactivity to Calcitonin Antibody (arrow) although greatest most para-follicular cells recognized as blue negative nucleus. **(C)** Thyroid gland section of (G3) highlighted a strong brown positive reactivity to Calcitonin-Antibody (arrow). **(D)** Thyroid gland section of (G4) signified mild reactivity with brown positive expression to Calcitonin-Antibody (arrow). (Hematoxylin & Eosin Stain, Magnification Power= x400 & Scale Bar= 50μm).

**Figure 5:**
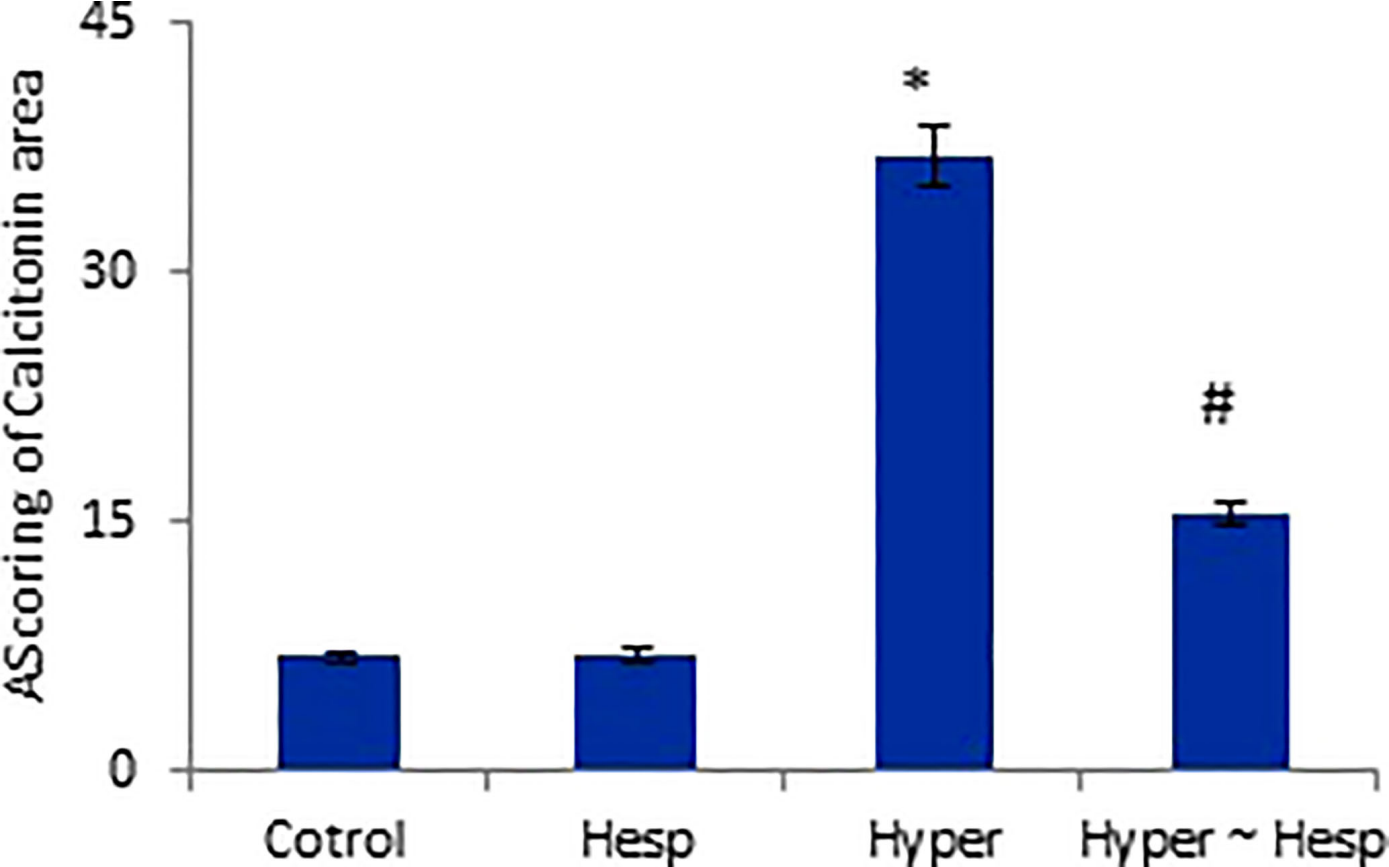
Scoring of Calcitonin area of healthy and hyperthyroidism treated animals’ groups as compared to control group. Data are presented as mean ± standard error, and they were subjected to one-way ANOVA followed by *post-hoc* test (Duncan) at p≤ 0.05. Symbol * is significantly different from control group, while symbol # is significantly different from hyperthyroidism group.

The results of the immunohistochemical analysis of thyroid gland sections showed that both normal and hesperidin-treated healthy animals expressed calcitonin-antibody in small number, while hyperthyroidism-modeled animals showed a robust brown positive reactivity to calcitonin-antibody, reflecting the high expression of calcitonin-antibody. Favorably, hyperthyroidism-hesperidin treated animals performed sharp improvement as it indicated lower reactivity towards calcitonin-antibody (**Figures 4A–D**, **5**).

## Discussion

Increases in thyroid hormones, as well as excessive production and secretion of free thyroid hormones (T3 and T4) which are circulating in the blood, are what cause hyperthyroidism, also known as an overactive thyroid gland (13). In pathological conditions such Graves’ disease and thyroid tumors, where the pituitary gland drives thyroid cells to produce additional hormones, causing a hyperthyroid state, the excessive levels of these hormones lead to some biochemical and clinical problems (18). The purpose of the current study was to assess the anti-hyperthyroidism, immunomodulatory, anti-inflammatory, antioxidant, and immunohistochemical effects of hesperidin against hyperthyroidism-modeled female rats. Hyperthyroidism-modeled animals showed a considerable increase in serum total T3, FT3, total T4, and FT3 levels along with a minor drop in serum TSH level; these results are consistent with those of Mannaa et al. (1) Gluvic et al. (19) and Meriem et al., (20). It has been stated that LT4 promotes thyroid activity and works primarily by inhibiting oxidative iodination within the thyroid gland to affect the synthesis of the thyroid hormones (thyroxin and triiodothyronine). Additionally, L-thyroxin interferes with peripheral T4 deiodination, which causes TSH to be produced outside of the thyroid gland (21). The anterior pituitary gland’s decreased production of TSH extends a negative feedback impact on the thyroid gland’s excessive production of T3 and T4. The anti-hyperthyroidism potential of hesperidin was demonstrated herein as it successfully reduced the levels of T3 and T4 post treating the hyperthyroidism-modeled animals; this effect may be attributed to hesperidin free radical-scavenging and antioxidant properties, which have been confirmed by the remarkable improvement in the oxidative status following hesperidin administration. Omar et al. (22) report that a hyperthyroidism-model revealed a rise in oxidative indicators (MDA and NO) associated with a decrease in the antioxidant batteries (GSH, GPx, CAT and SOD).

According to earlier research, hyperthyroidism causes liver apoptosis, which leads to hepatic dysfunction that accompanied by excessive release of extracellular free radicals, which in its turn lowers the body’s overall antioxidant capacity. Due to hyperthyroidism, MDA and NO levels were increased because thyroid hormones affect the amount of fat in rat tissues, promoting the production of reactive oxygen species (ROS), nitric oxide, and free radical formation. Lipid peroxidation (MDA), which is a good indicator for these activities, is also affected by thyroid hormones (1, 20, 23). According to Babu et al. (24), hyperthyroidism is a hypermetabolic illness that causes an increase in the generation of ROS, with SOD serving as the first line of defence.

The results of the current study showed that hesperidin post-treatment of hyperthyroidism rats reduced the MDA level, and created a substantial increase in CAT, GPx, and SOD activities as well as GSH level compared to their values of the untreated hyperthyroidism modeled group. These findings corroborate those of Mansour et al. (25); Omar et al. (22). Hesperidin’s ability to scavenge free radicals and inhibit lipid peroxidation in biological membranes is thought to be the cause of its modulatory activity against the disturbed oxidative stress (26).

Hesperidin has a substantial antioxidant activity, which has been demonstrated in numerous studies (27); in line with this, hesperidin lowered plasma NO levels *via* reducing induced oxidative stress. Scavenging reactive oxygen species and enhancing cellular antioxidant defence are the two main ways that hesperidin performs its antioxidant capabilities through (28). The main elements of the antioxidant defence system are GSH, SOD, and GPx; the lack of these elements can result in oxidative stress (29).

It was proposed that hyperthyroidism can cause the activation of proteins linked to inflammatory response, apoptosis, and hypertrophy (30). It was reported that NF-κB is activated by hyperthyroidism, which is crucial for controlling the genetic transcription and encoding of inflammatory cytokines including IL-10, IL-4, TNF-α, and IL-6 that, in turn, promote NO, lipid peroxidation and free radical generation (31–33). This mechanism is consistent with the findings of the current study which revealed a significant rise in TNF-α, IL-1β, IL-4, IL-6, IL-10 levels in the hyperthyroid group. Simsek et al. (34) recorded increases of IL-10, TNF-, IL-1β, and IL-6 levels in patients with hyperthyroidism. In both cellular and animal models, as well as in human umbilical vein endothelial cells, hesperidin’s anti-inflammatory action has been effectively demonstrated (35). Additionally, hesperidin was shown to reduce the production of IL-1β, IL-6, and TNF-β in a rat model of rheumatoid arthritis (36). The current findings thus verified that hesperidin’s powerful anti-inflammatory activity was linked to its ability to treat hyperthyroidism.

It was reported that the shape of the kidneys, glomerular filtration rate, renal hemodynamics, membrane transport, and sodium and water homeostasis are all impacted by thyroid hormones (37). Hyperthyroidism’s increases mitochondrial energy metabolism and decreases antioxidant enzymes of hepato-renal tissues, leading to excessive free radical production, and hepato-nephro damages, as consequence (37, 38).

Hesperidin’s hepato-nephroprotective action was demonstrated in the current study, as it effectively decreased the hyperthyroidism-related elevation of serum ALAT, ASAT, urea, and creatinine values. This effect could be attributed to stabilizing the hyperthyroidism-induced damage to the hepatic cellular membrane and protecting the hepatocyte and nephrites *via* its antioxidant effect and free radical scavenging activity (22, 39, 40).

The elevated serum LDL-c, triglyceride, and total cholesterol values here in hyperthyroidism group are consistent with those reported by many previous studies (1, 41). Likewise, Franke et al. (42) found that HDL-c level was significantly reduced in hyperthyroidism model rats.

The present study revealed that hesperidin markedly restored the hyperthyroidism-associated atherosclerotic disturbances; this anti-atherosclerotic effect agonists that of Kim and Lee (43) and Diekman et al. (44) and could be attributed to the rapid removal of chylomicron remnants from blood, which stimulates cholesterol ester transfer and lipoprotein lipase, increases LDL receptor, and enhances LDL receptor-mediated catabolism of LDL particles. Also, the reduction in triglycerides level runs in line with Kim and Lee (43) who interpreted that to increased hepatic triglyceride lipase activity, that enhanced cholesterol ester.

Both decreased peripheral insulin sensitivity and impaired insulin secretion are the factors contributing to the development of abnormal glucose tolerance in the hyperthyroid state (45). The clinical and experimental hyperthyroidism is often accompanied by abnormal glucose tolerance, which is attributed to mitochondrial oxidative damage caused by the excessive production of reactive nitrogen and oxygen species resulting from the overproduction of the thyroid hormones (46). In the current study, hesperidin was shown to be able to restore the elevated blood glucose level in hyperthyroid animals to a comparable level of control animals. Hesperidin is a flavonoid that can normalize blood sugar levels by affecting the functions of glucose transporter type-4 which stimulates the uptake of glucose in skeletal muscle and adipocytes, which may improve insulin resistance (47).

Our biochemical results were confirmed by the histopathological investigations of the thyroid gland tissue. Both control and hesperidin-treated normal animals showed normal thyroid architectures, and lowest reactivity against Calcitonin antibody. In contrast, hyperthyroidism animals performed deteriorated structure and number of thyroid follicles with a marked reactivity towards Calcitonin antibody; these observations run parallel to those of Mannaa et al. (48). Additionally, Nasikas et al., (49) reported that only Calcitonin immunostaining may be used to detect reactive, diffuse C-cell hyperplasia. In an efficient manner, hesperidin markedly succeeded in restoring the histological and immunohistochemical disturbances that accompanied the hyperthyroidism status; this was achieved by the renormalization of the histological architecture and the downregulation of the reactivity towards Calcitonin antibody close to the normal group; this improvement occurred probably by anti-inflammatory and anti-oxidative properties that preventing oxidative stress, and by ameliorating thyroid hormones. Man et al. (50) stated that hesperidin induced inhibition of p38 MAPK signaling pathway that in turn could contribute its attenuation of inflammation.

## Conclusion

The current study may lead to the conclusion that hesperidin is effective at modulating hyperthyroidism; this was evidenced by the pathophysiological, immunological, apoptotic, histopathological, and immunohistochemical improvements, as hesperidin has ability to reverse the disturbance in thyroid hormones and lessen its side effects proved successful in achieving this. Immune, inflammatory, apoptotic, and/or antioxidant regulatory processes may be how this effectiveness is mediated. To maximize the therapeutic efficacy of hesperidin, more research using various dosages is advised. The current study recommends using hesperidin to modulate hyperthyroidism and its associated complications.

## Data availability statement

The original contributions presented in the study are included in the article/supplementary material. Further inquiries can be directed to the corresponding authors.

## Ethics statement

The studies involving human participants were reviewed and approved by Al-Azhar University. Written informed consent for participation was not required for this study in accordance with the national legislation and the institutional requirements. The animal study was reviewed and approved by Al-Azhar University.

## Author contributions

All authors listed have made a substantial, direct, and intellectual contribution to the work, and approved it for publication.
